# Linking peripheral CD8^+^ single‐cell transcriptomic characteristics of mood disorders underlying with the pathological mechanism

**DOI:** 10.1002/ctm2.489

**Published:** 2021-07-19

**Authors:** Jing Lu, Lifeng Ma, Jiajun Jiang, Bochao Huang, Tingting Mou, Tingting Huang, Yi Xu, Ming Li, Lin Zhang, Xiaoping Han, Shaohua Hu

**Affiliations:** ^1^ Department of Psychiatry The First Affiliated Hospital Zhejiang University School of Medicine Hangzhou China; ^2^ The Key Laboratory of Mental Disorder Management in Zhejiang Province Hangzhou China; ^3^ Center for Stem Cell and Regenerative Medicine Zhejiang University School of Medicine Hangzhou China; ^4^ Key Laboratory of Animal Models and Human Disease Mechanisms of The Chinese Academy of Sciences and Yunnan Province Kunming Institute of Zoology Kunming China; ^5^ Netherlands Institute for Neuroscience An Institute of the Royal Netherlands Academy of Arts and Sciences Amsterdam The Netherlands; ^6^ Stem Cell Institute Zhejiang University Hangzhou China; ^7^ Zhejiang University Brain Research Institute Hangzhou China

Dear Editor,

Millions of individuals sustain mood disorders, including bipolar disorder (BD) and major depressive disorder (MDD), contributing a heavy burden to society.^1^ A previous study suggested dysfunction in the innate immune system underlies the pathophysiology of mood disorders.^2^ Meanwhile, certain components, such as natural killer (NK) cells and CD8+ lymphocytes, both belonging to killer lymphocytes, are dysfunctions of innate immunity and might participate in the pathogenesis of MDD and BD.^3^, ^4^

To study the characteristics of CD8+ T cells among BD, MDD, and healthy controls (HCs), 10x single‐cell RNA and T cell receptor (TCR) sequencing were applied to analyze the peripheral blood mononuclear cells from participants, including 4 BD patients, 4 MDD patients, and 4 HCs. T cells, expressing the TCR on the cell surface, received stimulation from pathogens and subsequently initiated an immune response.^5^ TIM‐3 has emerged as a critical regulator in T cells, and IL‐6, IL‐1β, and Caspase 3 have been linked to TIM‐3 activity. Hence, we examined plasma levels of these genes in patients with BD prior to and 1‐month after treatment with quetiapine.

A total of 14,098, 20,701, and 22,206 CD8+ T cells were isolated from patients with BD, MDD, and HCs, respectively. Twelve main clusters (Figure 1A) represented the different cell types (Figure 1B). The main CD8+ cell types were identified by comparing marker genes in various cell types (Figure 1C, Table S1). No significant differences in cell type clusters among the three groups (Figure 1D) were observed. The naiveness and cytotoxicity scores of each cell type were separately evaluated based on a predefined set of genes. Compared with that of other cell types, the naive score of NK cells expressing TIM‐3 was relatively low, while the effector score was the highest (Figures 1E and 1F), suggesting high expression levels of TIM‐3 from NK cells were related to activated cytotoxic phenotypes.

**FIGURE 1:**
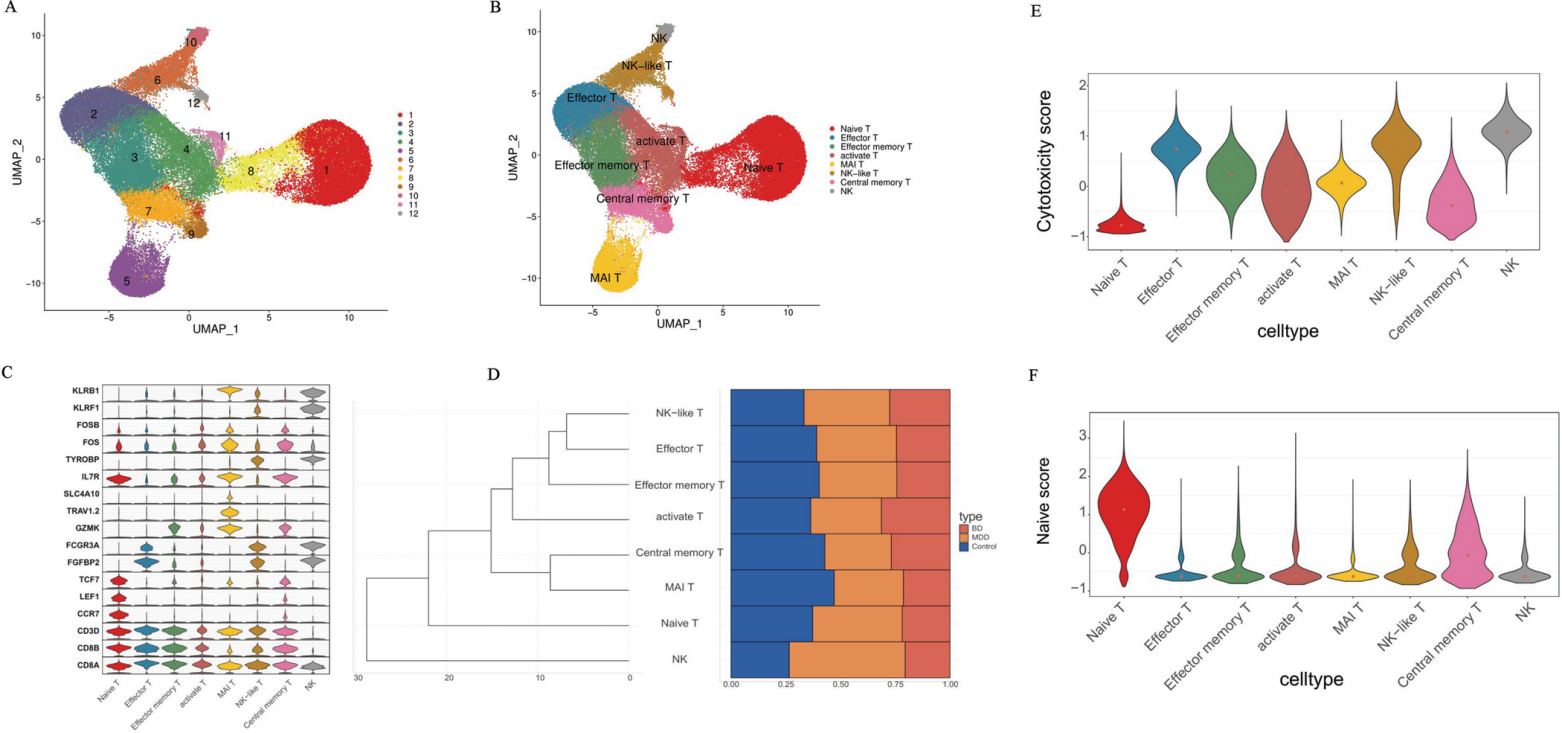
Single‐cell transcriptomes of CD8 + cells from BD, MDD, and controls. (A) A UMAP map of CD8+ cells from BD (*n* = 4), MDD (*n* = 4), and Control (*n* = 4). Cells are colored by 1–12 clusters. (B) A UMAP map of CD8+ cells from BD, MDD, and Control, and Cells are colored by cell types. (C) Violin plot showing expression comparison of marker genes in different cell types. (D) Cluster dendrogram shows clustering among cell types, and the bar plot shows cell fraction from BD, MDD, and Control in each cell type. (E and F) The naiveness score and cytotoxicity score of each cell type

We conducted gene expression analysis on CD8+ T cells from BD and HC patients; BD showed 262 upregulated and 403 downregulated genes (Figures 2A and 2B and Table S2), and MDD displayed 399 upregulated and 684 downregulated genes compared to those in HC (Figures 2E and 2F and Table S2). Moreover, compared to those in MDD, 466 genes were upregulated, and 253 genes were downregulated in the BD group (Figures 2C and 2D and Table S2). Among these, seven genes (Figure 2G) overlapped with BD susceptibility genes reported in a previous study,^6^ four genes (Figure 2H) overlapped with MDD risk genes previously reported in an RNA‐seq study.^7^ Furthermore, 17 genes (Figure 2I) overlapped with meta‐analysis data from one of the largest genome‐wide association studies for depression.^8^ Gene functional enrichment analysis was performed to explore biologically meaningful patterns in the transcriptome data by identifying characteristic pathways enriched in up‐ or downregulated genes from at least two cell types; we chose the most representative enrichment pathway terms, as shown in Tables S3‐S, 5. Additionally, we analyzed differentially expressed translational factors among BD, MDD, and HC (Figures S1‐S3).

**FIGURE 2:**
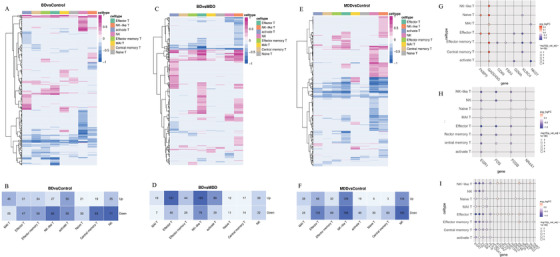
Differentially expressed genes between cells in clusters from BD, MDD, and controls. (A and B) Heatmap showing numbers of differentially expressed genes between BD and Control in each cell type. (C and D) Heatmap showing numbers of differentially expressed genes between MDD and Control in each cell type. (E and F) Heatmap showing numbers of differentially expressed genes between BD and MDD in each cell type. Red means genes upregulated, and blue denotes upregulated in the heatmap. Only the genes which absolute value of log fold change ≥ 0.25 and adjusted *p* value < 0.05 are marked with differentially expressed genes. (G) Changes of seven genes in each cell type which overlapped with BD susceptibility genes in previous studies,^6^ Red means upregulated, and blue means downregulated in BD. (H) Changes of four genes which were overlapped with MDD risk genes in an RNA‐seq study.^7^ Red means upregulated, and blue means downregulated in MDD. (I) Seventeen genes which were overlapped with a meta‐analyzed data from the largest genome‐wide association studies of depression.^8^ Red means upregulated, and blue means downregulated in MDD. Differentially expressed genes, see also Table S4

In our previous study,^1^ upregulation of TIM‐3 was noted in the cytotoxic T cells of BD patients where TIM‐3 was expressed in NK cells (cluster 10, Figure 3A). TIM‐3 (HAVCR2) gene expression within NK cells in BD was higher than in MDD and HC (*p* = 0.0014 and *p* = 0.031, respectively), while no significant changes were observed between MDD and HC (*p* = 0.41) group (Figure 3B). When comparing all cells, TIM‐3 gene expression was elevated in BD patients in comparison to MDD (*p* = 0.015) and HC (*p* < 0.001), whereas MDD patients had a higher TIM‐3 gene expression than that of HC (*p* = 0.022) (Figure 3C).

**FIGURE 3:**
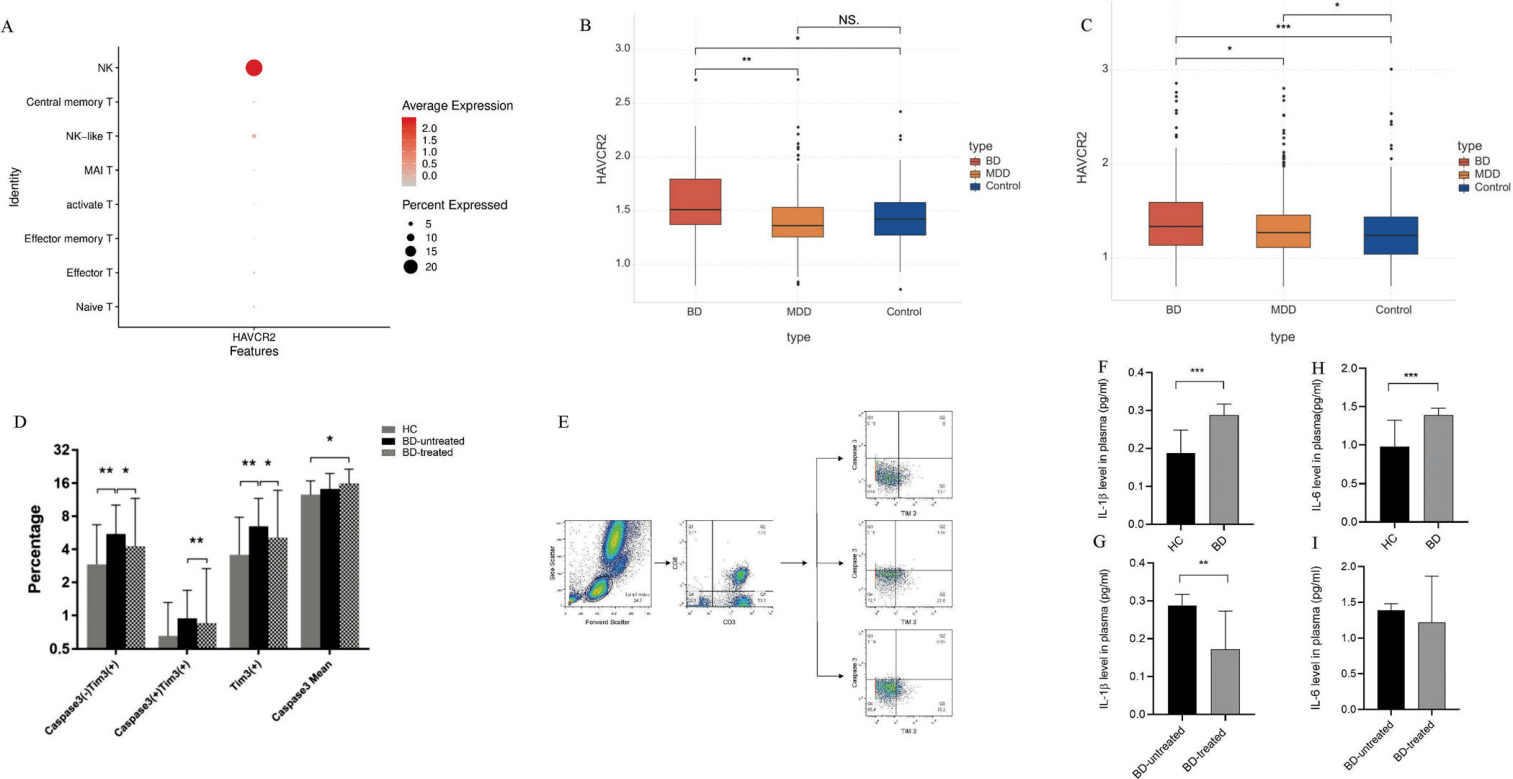
(A) HAVCR2 (TIM‐3) was expressed in NK cells (cluster 10). (B) Comparison of the expression level of HAVCR2 (TIM‐3) in NK cells among BD, MDD, and control. (C) The box plot shows the expression level of HAVCR2 between BD, MDD, and Control in all cells. (D) Expression levels of (Caspase3‐ TIM‐3+), (Caspase3+ TIM‐3+), TIM‐3+, and Mean TIM‐3+ on CD8+ monocytes in patients with BD and controls. *p < 0.05, **p < 0.01. (E) Flow cytometry gating strategy for the determination of TIM‐3 (+) on cytotoxic T cells monocytes. (F and G) Changes of plasma level of IL‐1β and IL‐6 in control, BD and BD after 4‐week treatment of quetiapine

Projection of TCR data onto the UMAP diagram showed naive T cells are mostly monoclonal, whereas cloned and amplified T cells are all concentrated in effector T cells (Figures S4A and S4B). The details and statistics on the use of V and J genes, as well as the *α* and *β* sequences, are displayed in Table S6. No significant differences were found in the proportion of monoclonal or polyclonal variety among the three groups (Figure S4C).

To further verify the correlation factor of TIM‐3 apoptotic pathway in BD, 31 BD patients (clinical information in Table S7) were treated with quetiapine (300 mg/d) for 4 weeks in the flow cytology study and compared with 31 HC. The proportion of (Caspase3‐ TIM‐3+) cells was upregulated in the BD patients compared to HC (*p* = 0.006) and decreased after quetiapine treatment (*p* = 0.021). The (Caspase3+ TIM‐3+) expression was decreased after treatment (*p* = 0.004, Figures 2D and 2E). Total cells expressing TIM‐3 were increased in BD (*p* = 0.008) and decreased after treatment compared to pre‐treatment (*p* = 0.016). However, Caspase3+ levels increased after treatment (*p* = 0.049). Moreover, in BD, plasma levels of IL‐1β and IL‐6 were significantly increased (*p* = 0.0004, *p* < 0.0001, respectively, Figures 3F‐3H); although IL‐1β decreased after treatment (*p* = 0.01, Figure 3G), no significant effect was observed in IL‐6 level (*p* = 0.134, Figure 3I).

In present study, single‐cell sequencing and flow cytometry verification showed that TIM‐3 expression in CD8+ T cells in BD group significantly increased in cell number and transcription level, compared with that in HC group and decreased after 4 weeks of quetiapine treatment. NK cells, mainly expressing TIM‐3, showed higher cytotoxicity. TCR analysis showed the proportion of cloned and amplified T cells in the BD and MDD groups was less than that in the HC group. The TCR diversity of BD was less than that of MDD patients.

In conclusion, this study provides the first insight into the cellular immune variables between BD, MDD, and HC indicating TIM‐3 expression might be involved in regulating the adaptive immune system by regulating T cell function and should be considered in the development of immune‐targeted therapeutic strategies.^9^ TIM‐3 might be a contributing factor in determining whether a T cell generates a dysfunctional or inflammatory response.^10^ Antipsychotics, which act as mood stabilizers, might be conducive to the reconstruction of the T cell response. Our study provided new evidences for immune dysfunction and new insights into immune checkpoint inhibitors of mood disorders underlying the pathological mechanism.

## CONFLICT OF INTEREST

The authors report no biomedical financial interests or potential conflict of interest.

## AUTHOR CONTRIBUTIONS

Jing Lu, Xiaoping Han, and Shaohua Hu were involved in the study design and the draft of manuscript. Lifeng Ma acquired and analyzed the data. Jiajun Jiang, Bochao Huang, and Tingting Mou were involved in the patient's collection and clinical assessment. Tingting Huang, Yi Xu, Ming Li, and Lin Zhang were involved in critical revision of the manuscript. Jing Lu, Lifeng Ma, Jiajun Jiang contribute equally to this work, and Shaohua Hu, Xiaoping Han are co‐correspongding authors. All authors read and approved the final manuscript.

## Supporting information

Supporting Information

Supporting Information

Supporting Information

Supporting Information

Supporting Information

Supporting Information

Supporting Information

Supporting Information

Supporting Information

Supporting Information

Supporting Information

Supporting Information
