# Supplementary material for: Linking peripheral CD8+ single‐cell transcriptomic characteristics of mood disorders underlying with the pathological mechanism
Source: Clin Transl Med. 2021 Jul 19;11(7):e489. doi: 10.1002/ctm2.489 (PMC8288008; doi:10.1002/ctm2.489)
Supplement: Supplementary file 3 — Supporting Information [file CTM2-11-e489-s006.docx]

|  | ***Category*** | ***Term*** | ***Description*** | ***LogP*** | ***Log(q-value)*** |
| --- | --- | --- | --- | --- | --- |
| Genes downregulated in BD | Reactome Gene Sets | R-HSA-9633012 | Response of EIF2AK4 (GCN2) to amino acid deficiency | -12.38893736 | -8.042682206 |
|  | KEGG Pathway | ko04380 | Osteoclast differentiation | -5.630407536 | -3.069482221 |
|  | GO Biological Processes | GO:0031960 | response to corticosteroid | -5.048660326 | -2.528479979 |
|  | Canonical Pathways | M60 | PID NFAT TFPATHWAY | -5.031668709 | -2.517922472 |
|  | Canonical Pathways | M183 | PID IL6 7 PATHWAY | -4.955464136 | -2.448058077 |
|  | GO Biological Processes | GO:0043618 | regulation of transcription from RNA polymerase II promoter in response to stress | -4.755983105 | -2.267060452 |
|  | GO Biological Processes | GO:0002764 | immune response-regulating signaling pathway | -4.47385627 | -2.002662384 |
|  | GO Biological Processes | GO:0002250 | adaptive immune response | -4.405578397 | -1.945813973 |
|  | GO Biological Processes | GO:0071480 | cellular response to gamma radiation | -4.032043953 | -1.61006809 |
|  | Canonical Pathways | M121 | PID MTOR 4PATHWAY | -2.956988995 | -0.750612932 |
|  | KEGG Pathway | ko05100 | Bacterial invasion of epithelial cells | -2.83570559 | -0.641738785 |
|  | GO Biological Processes | GO:0070848 | response to growth factor | -2.80149643 | -0.616609283 |
|  | GO Biological Processes | GO:0071396 | cellular response to lipid | -2.761416946 | -0.585423512 |
|  | GO Biological Processes | GO:0006417 | regulation of translation | -2.67551234 | -0.533377174 |
|  | GO Biological Processes | GO:0002366 | leukocyte activation involved in immune response | -2.291161434 | -0.27207909 |
|  | GO Biological Processes | GO:0001701 | in utero embryonic development | -2.259154192 | -0.255321724 |
|  | GO Biological Processes | GO:0045596 | negative regulation of cell differentiation | -2.124309056 | -0.161869273 |
|  | GO Biological Processes | GO:0097190 | apoptotic signaling pathway | -2.037002554 | -0.088050735 |
| Genes upregulate |  |  |  |  |  |

**Supplementary Table 3: Non-redundant lists of pathways enriched in differentially expressed genes between BD and control**

in BD

|  | GO Biological Processes | GO:0002250 | adaptive immune response | -9.237101652 | -4.895251827 |
| --- | --- | --- | --- | --- | --- |
|  | Reactome Gene Sets | R-HSA-163200 | Respiratory electron transport, ATP synthesis by chemiosmotic coupling, and heat production by uncoupling proteins. | -5.827717982 | -1.82943493 |
|  | KEGG Pathway | ko04514 | Cell adhesion molecules (CAMs) | -5.508124553 | -1.82943493 |
|  | GO Biological Processes | GO:0060968 | regulation of gene silencing | -2.705176341 | -0.314569281 |
|  | GO Biological Processes | GO:0098542 | defense response to other organism | -2.56329932 | -0.221449496 |

**Abbreviations: BD, bipolar disorder; Reported q-values derived by false discovery rate method (Benjamini-Hochberg).**
