# Supplementary material for: Linking peripheral CD8+ single‐cell transcriptomic characteristics of mood disorders underlying with the pathological mechanism
Source: Clin Transl Med. 2021 Jul 19;11(7):e489. doi: 10.1002/ctm2.489 (PMC8288008; doi:10.1002/ctm2.489)
Supplement: Supplementary file 4 — Supporting Information [file CTM2-11-e489-s005.docx]

**Supplementary Table 4: Non-redundant lists of pathways enriched in differentially expressed genes between BD and MDD**

|  |  | ***Category*** | ***Term*** | ***Description*** | ***LogP*** | ***Log(q-value)*** |
| --- | --- | --- | --- | --- | --- | --- |
| Genes downregulated in BD than MDD | | GO Biological Processes | GO:0002250 | adaptive immune response | -5.424091973 | -2.509200588 |
|  |  | GO Biological Processes | GO:0034109 | homotypic cell-cell adhesion | -4.745530342 | -1.967476917 |
|  |  | GO Biological Processes | GO:0043900 | regulation of multi-organism process | -3.949324881 | -1.343432421 |
|  |  | GO Biological Processes | GO:0060759 | regulation of response to cytokine stimulus | -3.37964757 | -0.865901333 |
|  |  | GO Biological Processes | GO:0052548 | regulation of endopeptidase activity | -2.967654155 | -0.540477098 |
|  |  | GO Biological Processes | GO:0043270 | positive regulation of ion transport | -2.781188205 | -0.389175565 |
|  |  | Reactome Gene Sets | R-HSA-198933 | Immunoregulatory interactions between a Lymphoid and a non-Lymphoid cell | -2.767443614 | -0.380229857 |
|  |  | GO Biological Processes | GO:0007420 | brain development | -2.643506571 | -0.292886616 |
|  |  | Reactome Gene Sets | R-HSA-5683057 | MAPK family signaling cascades | -2.568987198 | -0.235569274 |
|  |  | GO Biological Processes | GO:0002250 | adaptive immune response | -5.424091973 | -2.509200588 |
|  |  | GO Biological Processes | GO:0034109 | homotypic cell-cell adhesion | -4.745530342 | -1.967476917 |
| Genes upregulated in BD than MDD | | Canonical Pathways | M167 | PID AP1 PATHWAY | -9.53464 | -6.895954944 |
|  |  | KEGG Pathway | ko04210 | Apoptosis | -8.46613 | -5.868059309 |
|  |  | CORUM | CORUM:178 | Respiratory chain complex I (holoenzyme), mitochondrial | -7.74584 | -5.198922834 |
|  |  | GO Biological Processes | GO:1902107 | positive regulation of leukocyte differentiation | -6.66287 | -4.197432581 |
|  |  | Reactome Gene Sets | R-HSA-449147 | Signaling by Interleukins | -6.63695 | -4.177188177 |
|  |  | Reactome Gene Sets | R-HSA-9031628 | NGF-stimulated transcription | -6.3939 | -3.95073798 |
|  |  | KEGG Pathway | ko05321 | Inflammatory bowel disease (IBD) | -5.27175 | -2.99368499 |
|  |  | Reactome Gene Sets | R-HSA-9663891 | Selective autophagy | -4.80138 | -2.578975083 |
|  |  | GO Biological Processes | GO:0097190 | apoptotic signaling pathway | -4.59678 | -2.405864335 |
|  |  | GO Biological Processes | GO:0001906 | cell killing | -4.19492 | -2.10151578 |
|  |  | GO Biological Processes | GO:0043618 | regulation of transcription from RNA polymerase II promoter in response to stress | -4.17958 | -2.091007266 |
|  |  | GO Biological Processes | GO:0070555 | response to interleukin-1 | -3.76325 | -1.743333336 |
|  |  | GO Biological Processes | GO:0002274 | myeloid leukocyte activation | -3.59633 | -1.602253073 |
|  |  | GO Biological Processes | GO:0000302 | response to reactive oxygen species | -3.50183 | -1.530321705 |
|  |  | KEGG Pathway | ko04064 | NF-kappa B signaling pathway | -3.29309 | -1.358451476 |
|  |  | GO Biological Processes | GO:0071674 | mononuclear cell migration | -3.24206 | -1.329901615 |
|  |  | GO Biological Processes | GO:0061640 | cytoskeleton-dependent cytokinesis | -3.20898 | -1.306770492 |

**Abbreviations: BD, bipolar disorder, MDD, major depressive disorder; Reported q-values derived by false discovery rate method (Benjamini-Hochberg).**
