# Supplementary material for: Linking peripheral CD8+ single‐cell transcriptomic characteristics of mood disorders underlying with the pathological mechanism
Source: Clin Transl Med. 2021 Jul 19;11(7):e489. doi: 10.1002/ctm2.489 (PMC8288008; doi:10.1002/ctm2.489)
Supplement: Supplementary file 5 — Supporting Information [file CTM2-11-e489-s002.docx]

**Supplementary Table 5: Non-redundant lists of pathways enriched in differentially expressed genes between MDD and control**

|  | ***Category*** | ***Term*** | ***Description*** | ***LogP*** | ***Log(q-value)*** |
| --- | --- | --- | --- | --- | --- |
| Genes downregulated in MDD | GO Biological Processes | GO:0006413 | translational initiation | -10.25567002 | -6.030692544 |
|  | CORUM | CORUM:178 | Respiratory chain complex I (holoenzyme), mitochondrial | -8.367089469 | -4.817271343 |
|  | Reactome Gene Sets | R-HSA-449147 | Signaling by Interleukins | -7.365348731 | -4.380821418 |
|  | KEGG Pathway | ko04210 | Apoptosis | -7.116914144 | -4.262020688 |
|  | Canonical Pathways | M167 | PID AP1 PATHWAY | -6.923686912 | -4.10891068 |
|  | GO Biological Processes | GO:0070555 | response to interleukin-1 | -6.544288867 | -3.888229798 |
|  | GO Biological Processes | GO:0002274 | myeloid leukocyte activation | -6.062315726 | -3.52224055 |
|  | Reactome Gene Sets | R-HSA-166058 | MyD88:MAL(TIRAP) cascade initiated on plasma membrane | -6.008907008 | -3.495160771 |
|  | GO Biological Processes | GO:0002250 | adaptive immune response | -5.740855713 | -3.324019489 |
|  | GO Biological Processes | GO:0002228 | natural killer cell mediated immunity | -5.720847248 | -3.314111351 |
|  | GO Biological Processes | GO:0043618 | regulation of transcription from RNA polymerase II promoter in response to stress | -5.605992341 | -3.228220141 |
|  | GO Biological Processes | GO:1903706 | regulation of hemopoiesis | -5.368565396 | -3.051850481 |
|  | KEGG Pathway | ko04612 | Antigen processing and presentation | -5.364681875 | -3.051850481 |
|  | Reactome Gene Sets | R-HSA-9663891 | Selective autophagy | -5.236186818 | -2.958117531 |
|  | GO Biological Processes | GO:0006979 | response to oxidative stress | -4.99906307 | -2.792523052 |
|  | Reactome Gene Sets | R-HSA-5687128 | MAPK6/MAPK4 signaling | -4.998899115 | -2.792523052 |
|  | GO Biological Processes | GO:0071480 | cellular response to gamma radiation | -4.66572654 | -2.544780672 |
|  | GO Biological Processes | GO:0045948 | positive regulation of translational initiation | -4.607587147 | -2.514185028 |
| Genes upregulated in MDD | GO Biological Processes | GO:0002250 | adaptive immune response | -9.66775 | -5.32149 |
|  | GO Biological Processes | GO:0034109 | homotypic cell-cell adhesion | -6.27839 | -2.26531 |
|  | KEGG Pathway | ko04658 | Th1 and Th2 cell differentiation | -6.13444 | -2.26531 |
|  | GO Biological Processes | GO:0030155 | regulation of cell adhesion | -5.89286 | -2.24558 |
|  | KEGG Pathway | hsa05163 | human cytomegalovirus infection | -5.77952 | -2.21141 |
|  | KEGG Pathway | ko04670 | Leukocyte transendothelial migration | -5.58757 | -2.13311 |
|  | KEGG Pathway | ko05100 | Bacterial invasion of epithelial cells | -5.20902 | -1.97671 |
|  | GO Biological Processes | GO:0072657 | protein localization to membrane | -4.86203 | -1.81251 |
|  | GO Biological Processes | GO:0045055 | regulated exocytosis | -4.77957 | -1.75553 |
|  | Canonical Pathways | M124 | PID CXCR4 PATHWAY | -4.60585 | -1.72341 |
|  | Reactome Gene Sets | R-HSA-9675108 | Nervous system development | -4.57005 | -1.72341 |
|  | GO Biological Processes | GO:0019722 | calcium-mediated signaling | -3.94981 | -1.41273 |
|  | Canonical Pathways | M23 | PID WNT NONCANONICAL PATHWAY | -3.75736 | -1.3032 |
|  | Canonical Pathways | M277 | PID INTEGRIN A4B1 PATHWAY | -3.717032239 | -1.268404181 |
|  | Canonical Pathways | M159 | PID AMB2 NEUTROPHILS PATHWAY | -3.43468483 | -1.101133209 |
|  | Reactome Gene Sets | R-HSA-6783783 | Interleukin-10 signaling | -3.258920334 | -0.99184643 |
|  | KEGG Pathway | ko04650 | Natural killer cell mediated cytotoxicity | -2.95475169 | -0.796017262 |

**Abbreviations: MDD, major depressive disorder; Reported q-values derived by false discovery rate method (Benjamini-Hochberg).**
