# Supplementary material for: Linking peripheral CD8+ single‐cell transcriptomic characteristics of mood disorders underlying with the pathological mechanism
Source: Clin Transl Med. 2021 Jul 19;11(7):e489. doi: 10.1002/ctm2.489 (PMC8288008; doi:10.1002/ctm2.489)
Supplement: Supplementary file 7 — Supporting Information [file CTM2-11-e489-s007.docx]

**Supplementary Table 7:. clinical evaluation information from Flow cytometry demographic study**

|  | **BD-pretreatment(N=31)**  **quetiapine（300mg/d）** | **Post-treatment(N=31)** | **Control (N=31)** | **P（BD vs CTR）** |
| --- | --- | --- | --- | --- |
| Age (years) | 22.51±7.75 | / | 27.63±7.684 | 0.879 |
| Sex(M/F) | 20/11 | / | 18/13 | 0.666 |
| Education | 12.29±3.13 | / | 14.88±1.844 | 0.160 |
| Age of onset | 17.22±4.86 | / | / | / |
| Total course of disease | 3.83±4.51 | / | / | / |
| HAMD | 28.74±12.11 | 16.28 ±10.15 | 2.17±2.48 | <0.001 |
| HAMA | 23.66±10.15 | 11.80±8.50 | 1.48±2.14 | <0.001 |
| MADRS | 27.30±12.31 | 13.16±9.69 | 12.13±6.62 | <0.001 |

**Abbreviations: BD, bipolar disorder; CTR, control; HAMD, Hamilton Depression Scale，HAMA, Hamilton Anxiety Scale; MADRS, Montgomery-Asberg Depression Rating Scale**
