# Supplementary material for: Linking peripheral CD8+ single‐cell transcriptomic characteristics of mood disorders underlying with the pathological mechanism
Source: Clin Transl Med. 2021 Jul 19;11(7):e489. doi: 10.1002/ctm2.489 (PMC8288008; doi:10.1002/ctm2.489)
Supplement: Supplementary file 8 — Supporting Information [file CTM2-11-e489-s003.docx]

Supplementary Figures:


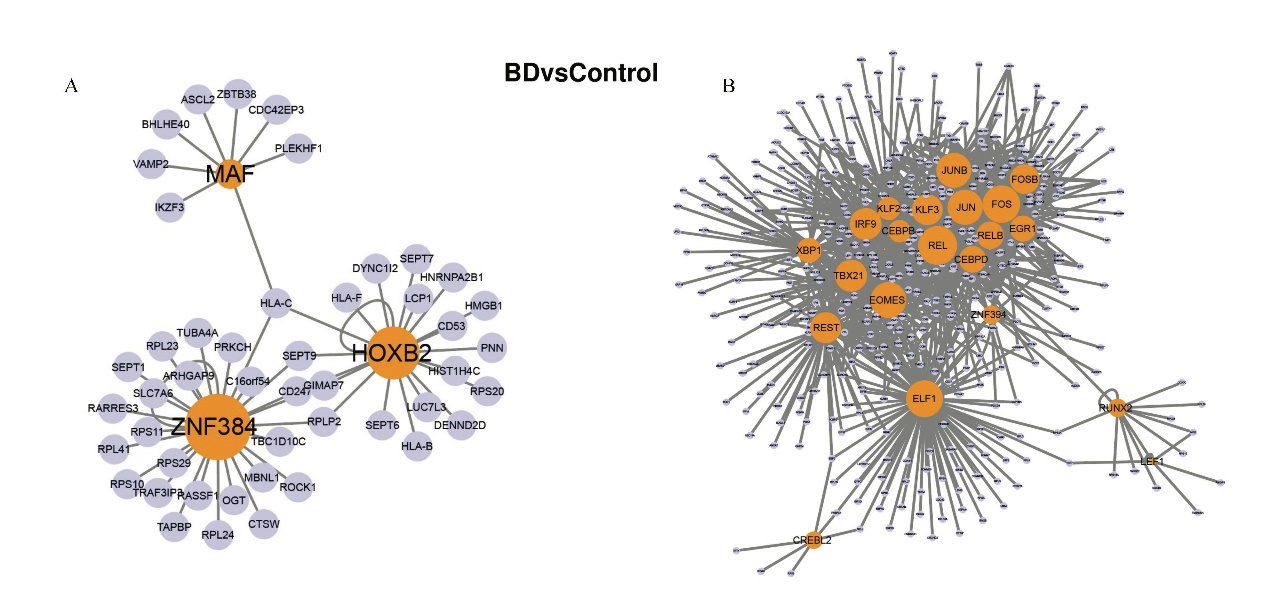


Supplementary Figure 1: Differentially expressed translational factors (TF) between BD and Control, (A) orange dots showing highly active TF in BD, (B) orange dots showing highly active TF in Control. Note: purple dots showing the differentially expressed genes between BD and Control.


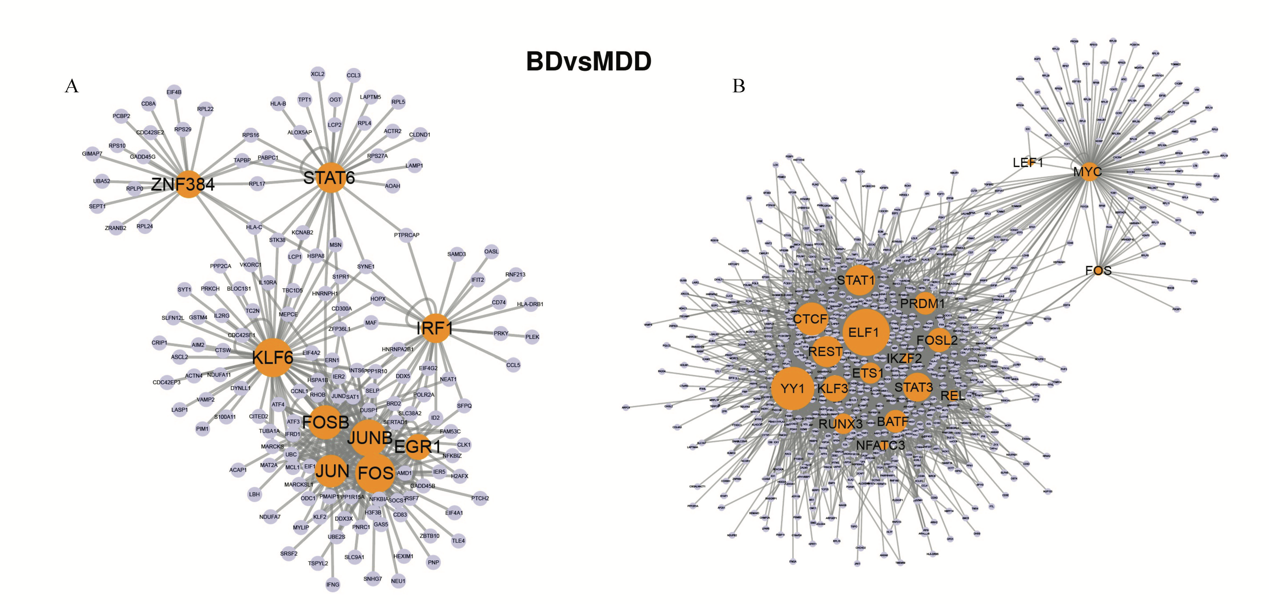


Supplementary Figure 2: Differentially expressed translational factors (TF) between BD and MDD, (A) orange dots showing highly active TF in BD, (B) orange dots showing highly active TF in MDD. Note: purple dots showing the differentially expressed genes between BD and MDD.


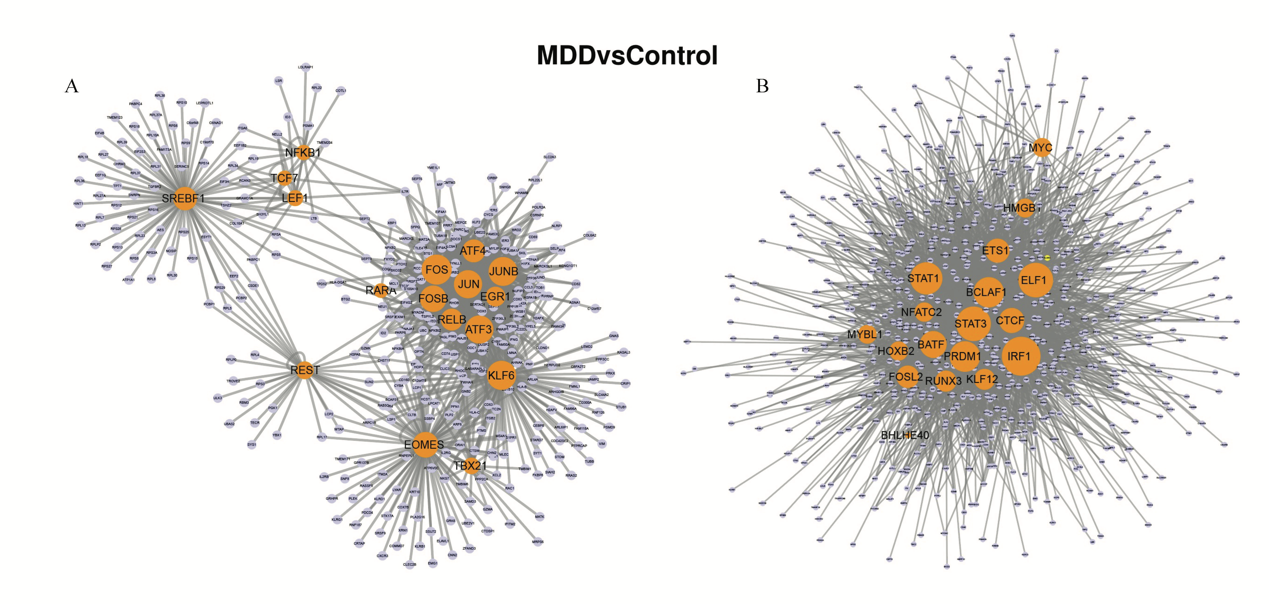


Supplementary Figure 3: Differentially expressed translational factors (TF) between MDD and Control, (A) orange dots showing highly active TF in MDD, (B) orange dots showing highly active TF in Control. Note: purple dots showing the differentially expressed genes between BD and MDD.


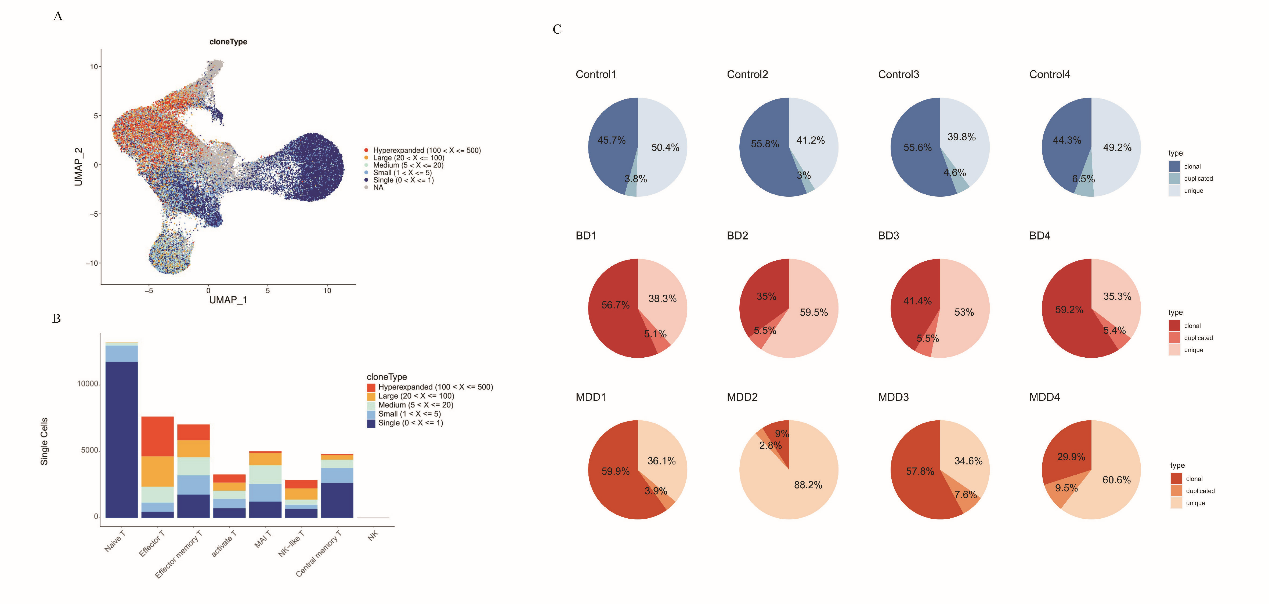


Supplementary Figure 4 (A): Projection of TCR data onto UMAP diagram (B): Bar plot shows the distribution of clone type in each cell type. (C) The pie charts shows the percentage of T cells for each BD, MDD patient, control found in clones of increasing size. Unique (n = 1), duplicated (n = 2) and the clonal (shared by at least 3 cells in a given cell population, n ≥3) are labeled with different colors.
