# Supplementary figures and images for: Linking peripheral CD8+ single‐cell transcriptomic characteristics of mood disorders underlying with the pathological mechanism
Source: Clin Transl Med. 2021 Jul 19;11(7):e489. doi: 10.1002/ctm2.489 (PMC8288008; doi:10.1002/ctm2.489)

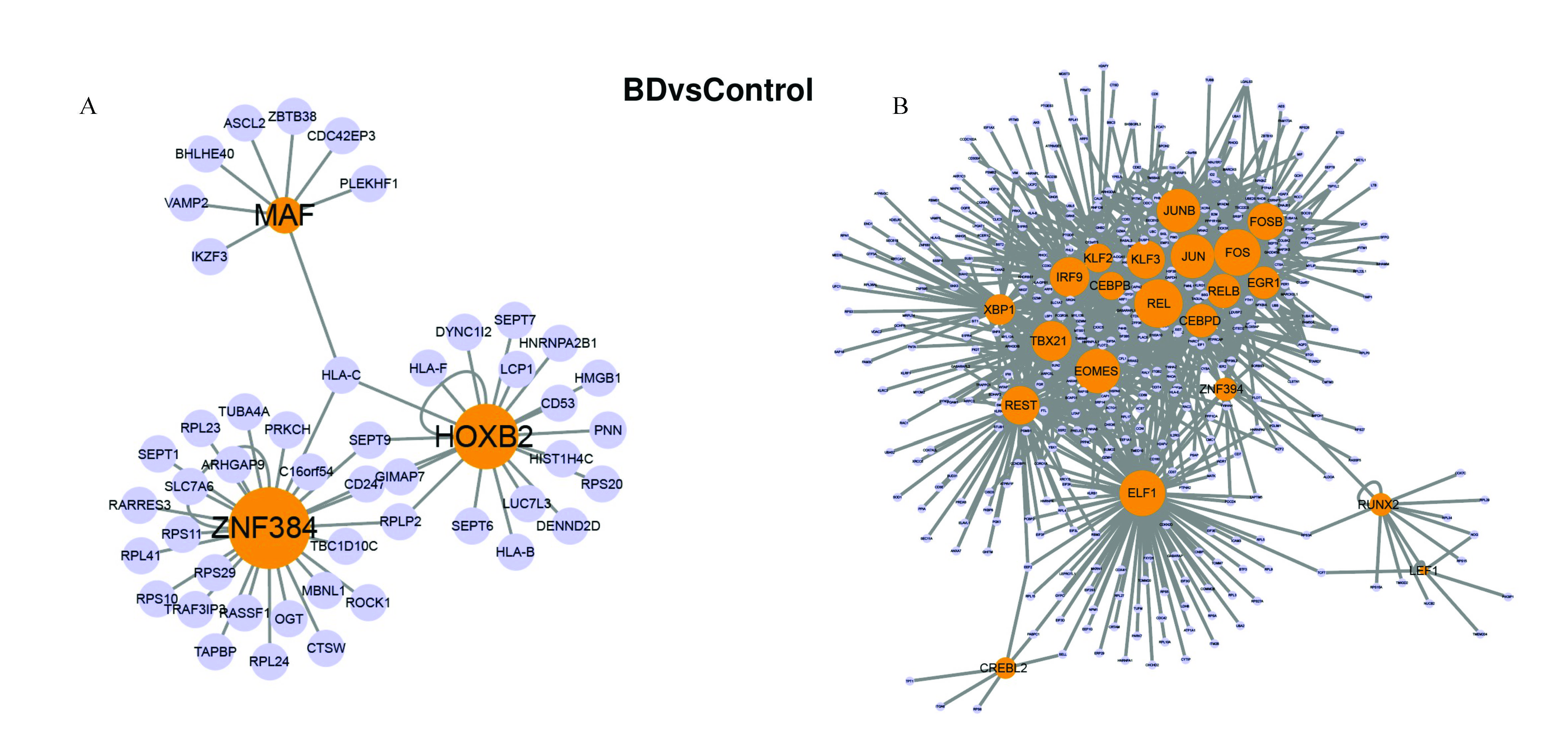

Supplement: Supplementary file 9 — Supporting Information [file CTM2-11-e489-s001.jpg]

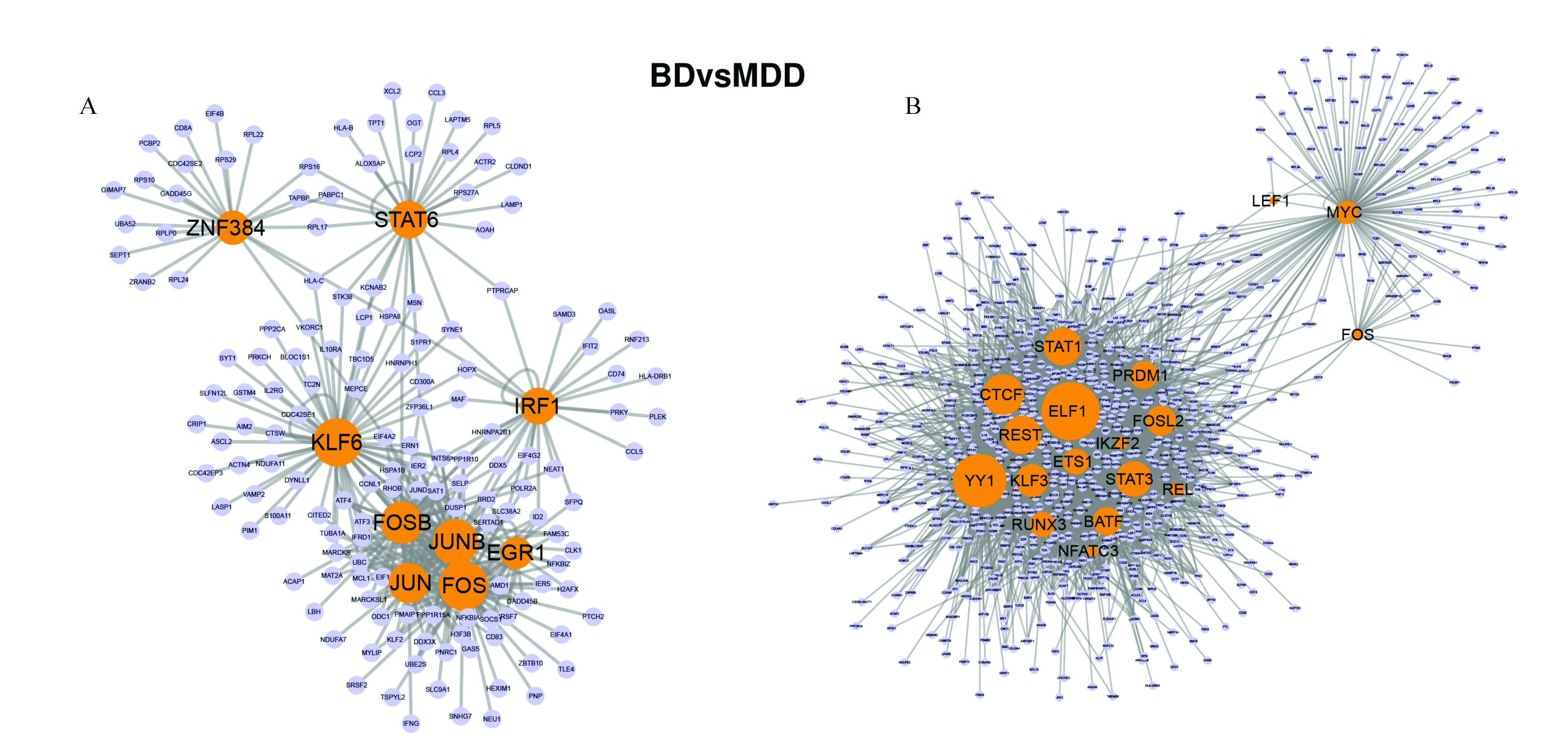

Supplement: Supplementary file 10 — Supporting Information [file CTM2-11-e489-s004.jpg]

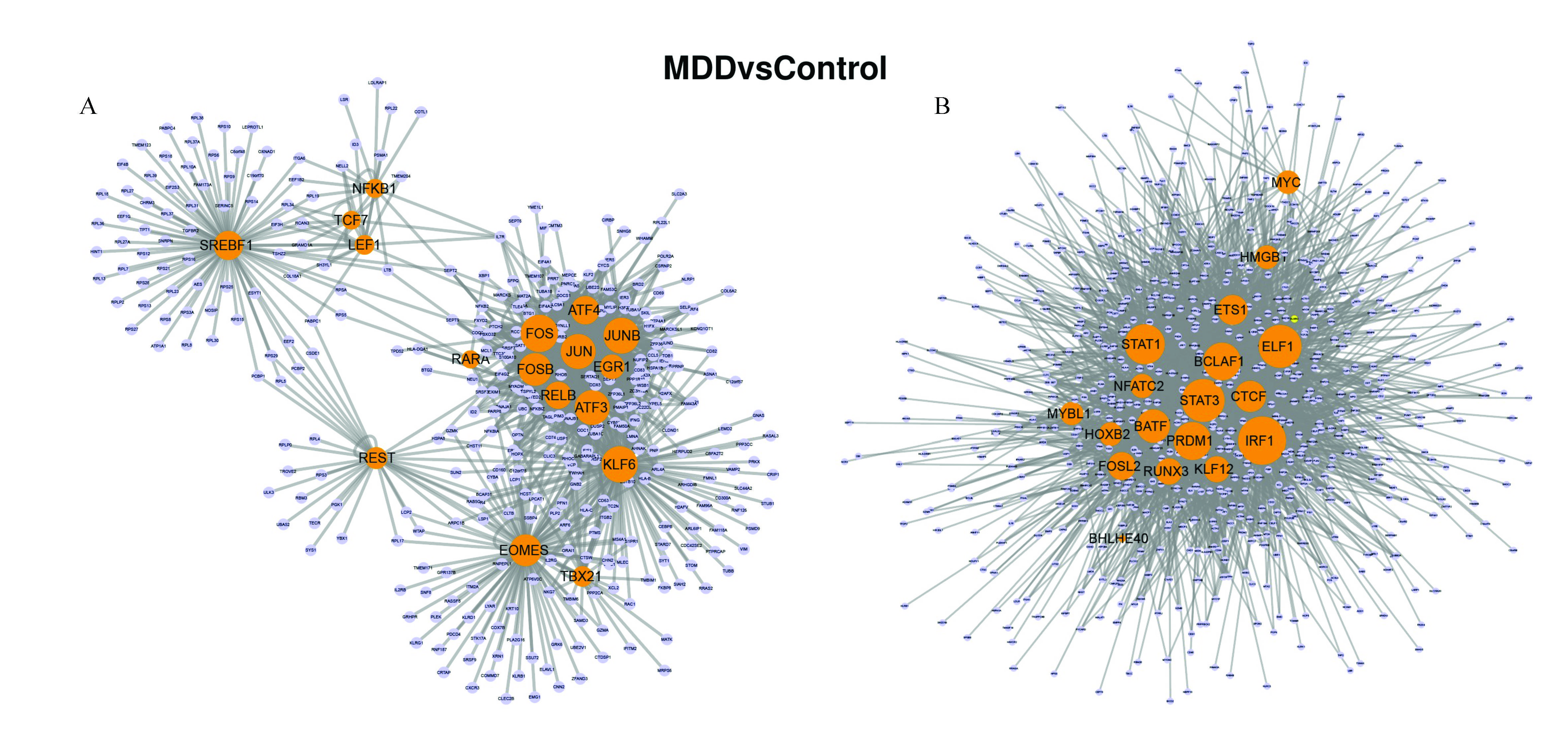

Supplement: Supplementary file 11 — Supporting Information [file CTM2-11-e489-s008.jpg]

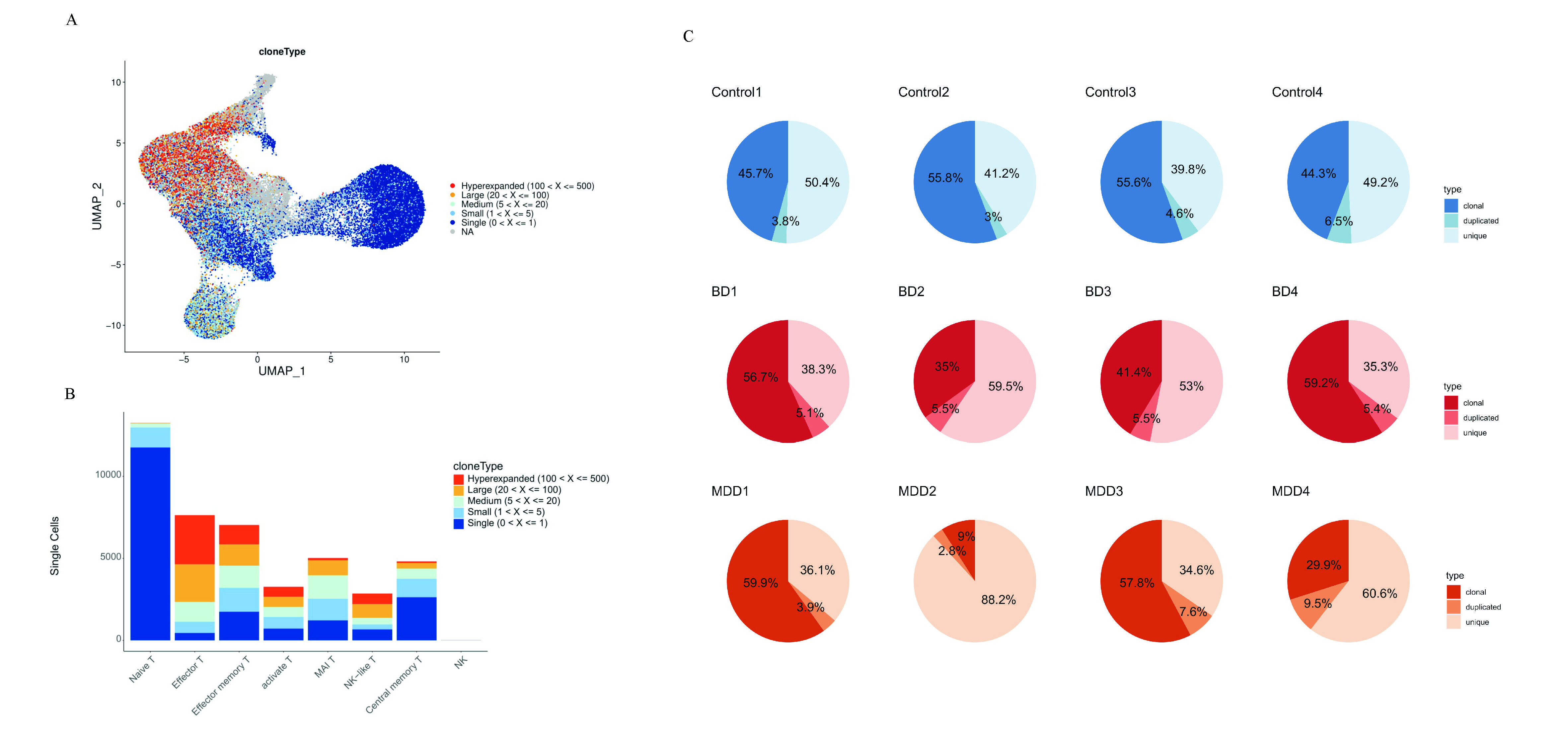

Supplement: Supplementary file 12 — Supporting Information [file CTM2-11-e489-s011.jpg]
